# Supplementary material for: Impact of forest disturbance on microarthropod communities depends on underlying ecological gradients and species traits
Source: PeerJ. 2023 Oct 5;11:e15959. doi: 10.7717/peerj.15959 (PMC10560493; doi:10.7717/peerj.15959)
Supplement: Supplemental Information 1 [file peerj-11-15959-s001.docx]

**SUPPLEMENTARY MATERIALS - APPENDICES**

**Impact of forest disturbance on microarthropod communities depends on underlying ecological gradient and species traits**

Davide Nardi*, Diego Fontaneto, Matteo Girardi, Isaac Chini, Daniela Bertoldi, Roberto Larcher, Cristiano Vernesi

**Appendix A – Sampling design**

**
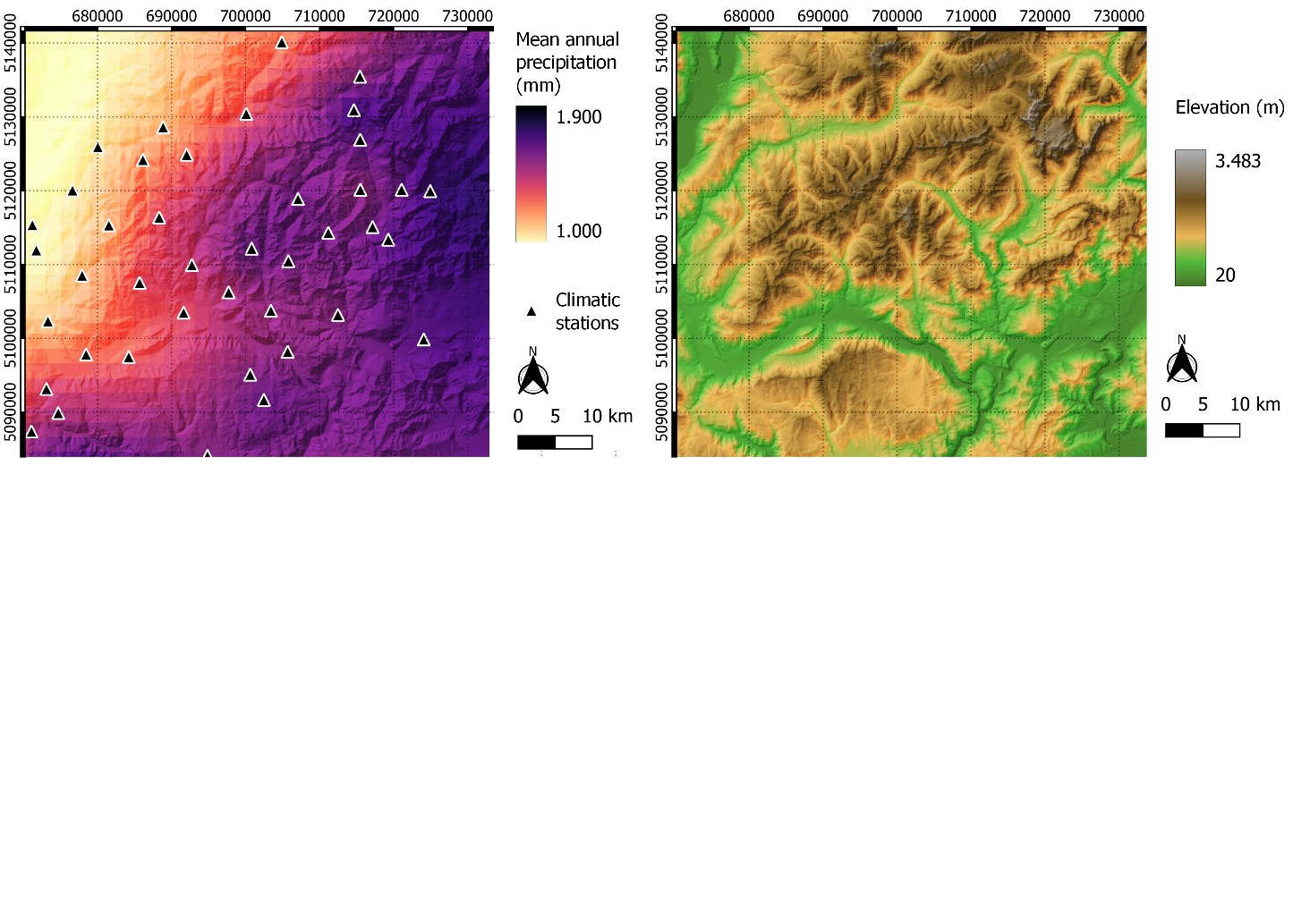
**

**Fig. A.1 -** Large-scale ecological gradients were used for sampling site designation. Mean annual precipitation raster was computed with kriging interpolation using climate station data. Source elevational data: www. land.copernicus.eu.

**
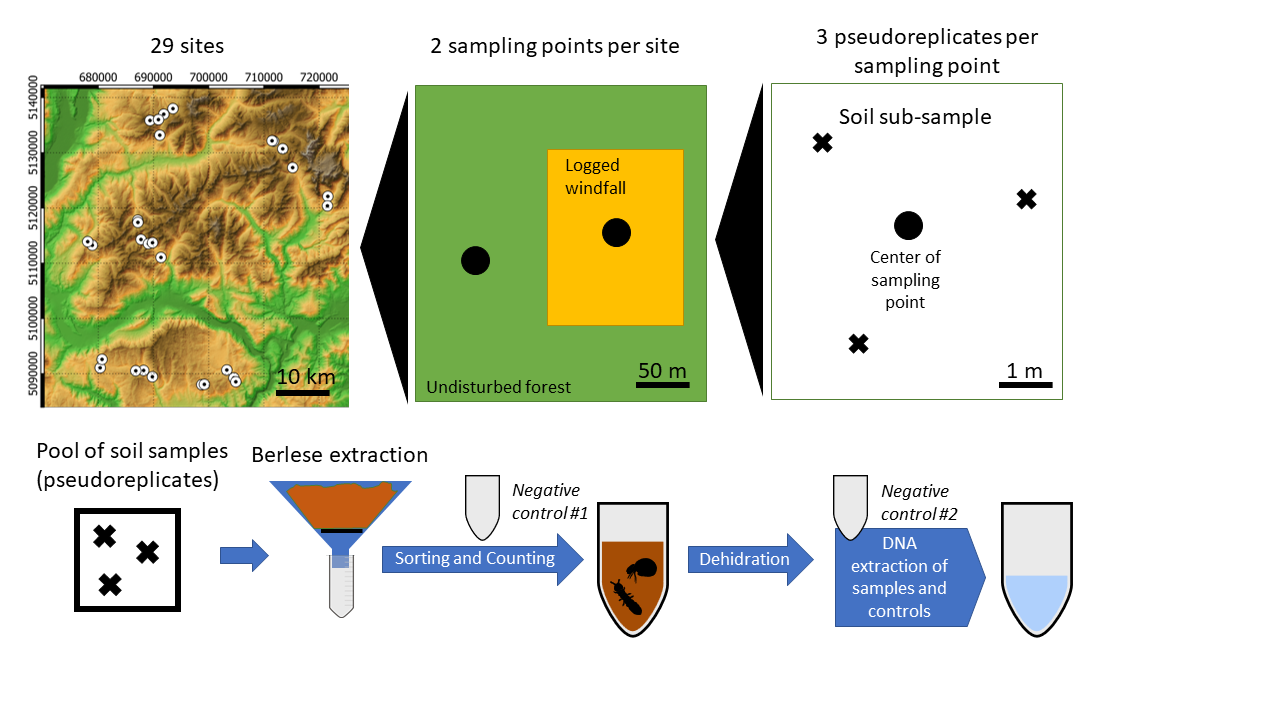
**

**Fig. A.2 –** Graphical scheme of sampling design and main steps of the lab protocol until PCR. Source elevational data: www. land.copernicus.eu.

**Appendix B – taxonomic resolution OUT and completeness of data**

Please refer to the online available data of this paper for more details on results of ASAP algorithm.

**Fig. B.1** – Taxonomic resolution achievable for springtail OTUs


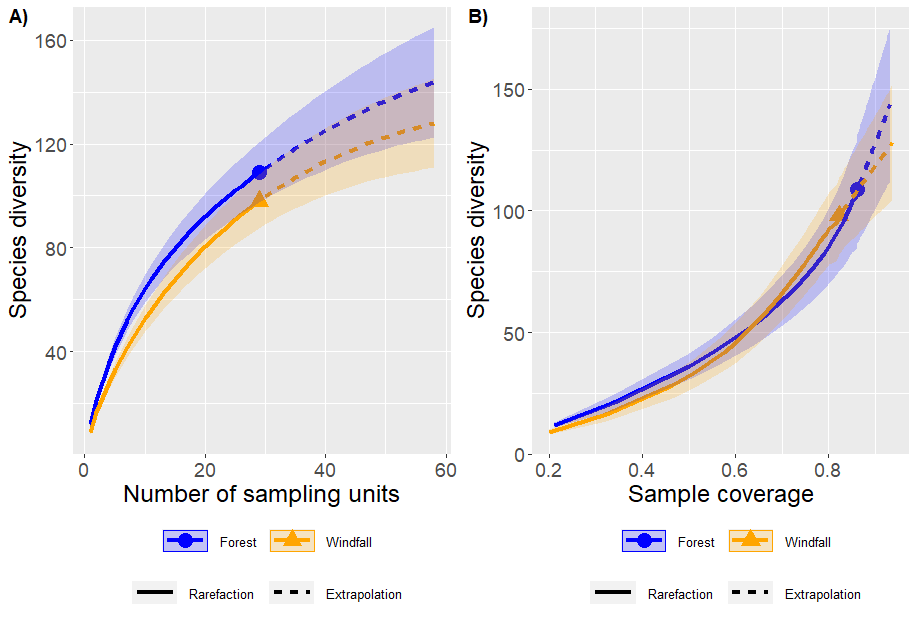


**Fig. B.2** - Rarefaction curves and extrapolation of cumulative species diversity for springtails

**Fig. B.3** – Taxonomic resolution achievable for soil mite OTUs


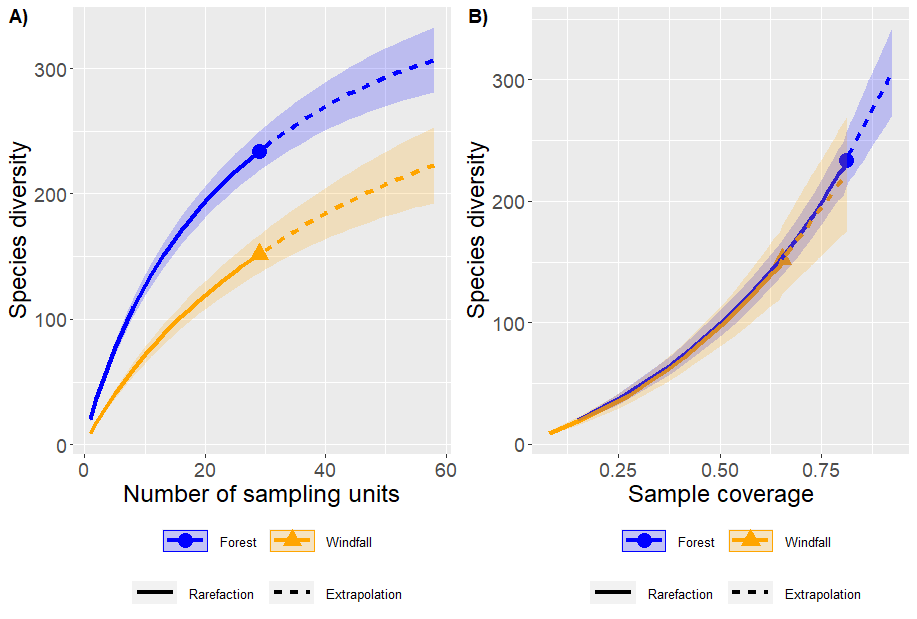


**Fig. B.4** - Rarefaction curves and extrapolation of cumulative species diversity for mites**Appendix C - Moss cover**

For each subplot (pseudo-replicates), we visually estimated moss coverage. Then, for each sampling point the average value was computed and used as environmental variable (expressed as percentage). We assessed moss cover loss between forest and windfall using linear mixed effect models with package lme4 version 1.27.1 (Bates et al., 2015) in R. We used moss cover as response variable, habitat as explanatory variable, and pair ID as random effect. We found that moss cover was overall reduced in windfalls (P value < 0.0001).


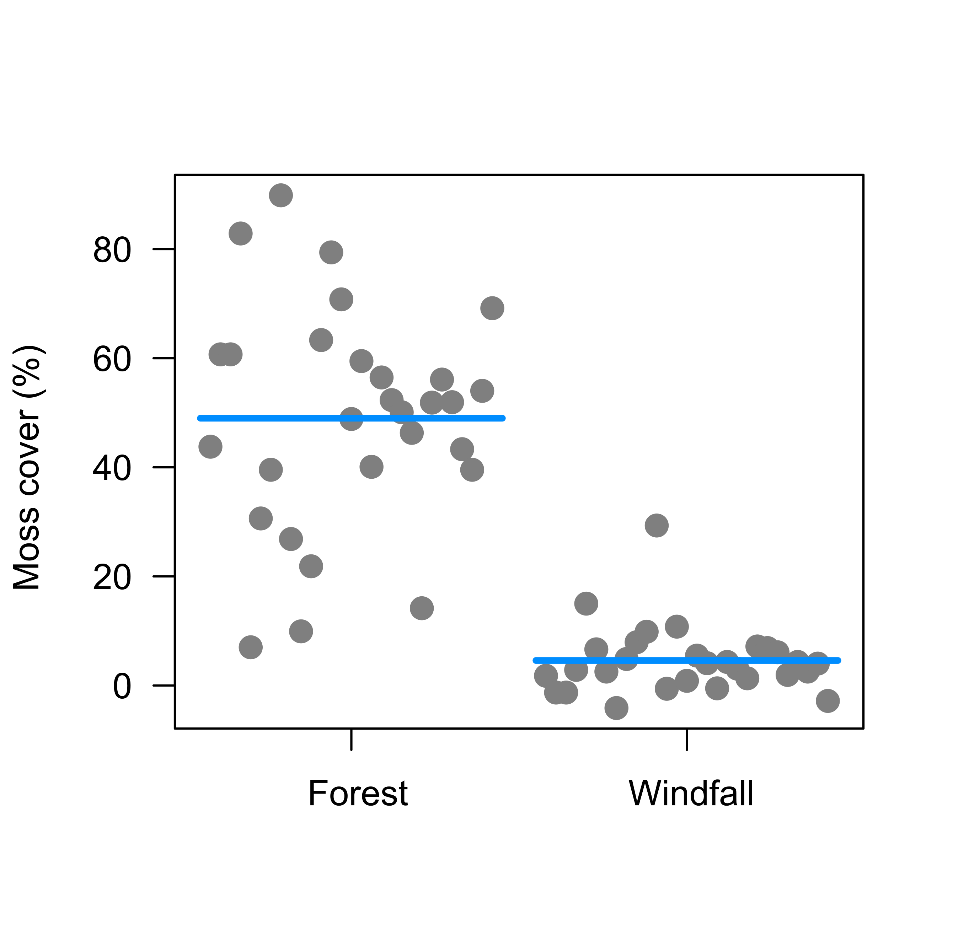


Fig C.1 - Moss cover loss due to habitat change (windstorm disturbance).

For springtails, we ran model using moss cover instead of habitat type strictly focusing on the role of the moss. Results are similar to those presented in the main text.

Model formula: Species richness ~ trophic guild * moss cover + random: ID nested in pair nested in geographic zone.

Table C.1 - Anova-table results of interaction between trophic guild and moss cover for springtails, with F and P values**.** Number of asterisks refer to significance levels: * = P < 0.05, *** = P < 0.001.

| **Springtails** | | | |
| --- | --- | --- | --- |
|  | **F** | **P** |  |
| Trophic guild | 41.67 | <0.0001 | *** |
| Moss cover | 0.60 | 0.4439 |  |
| Trophic guild : moss cover | 5.80 | 0.0009 | *** |


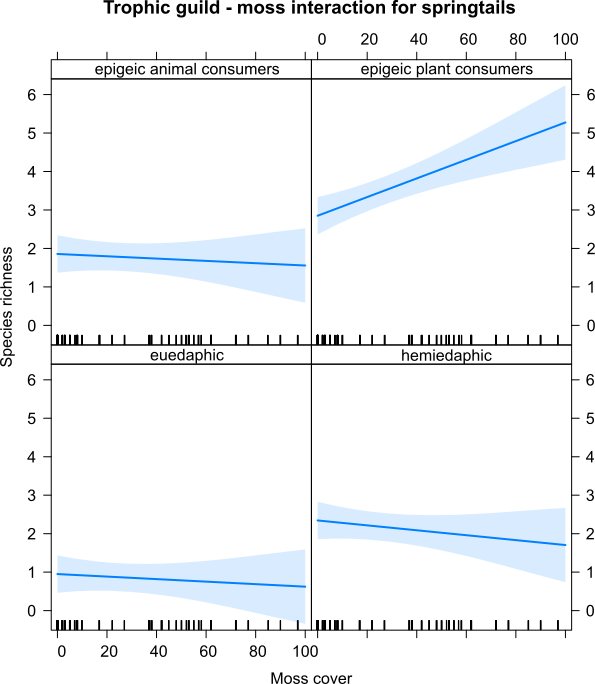


Fig. C.2 – Effect plot of model testing the interaction between trophic guild and moss cover for springtails.

**References**

Bates, D., Mächler, M., Bolker, B., & Walker, S. (2015). Fitting linear mixed-effects models using lme4. *Journal of Statistical Software*, *67*(1). https://doi.org/10.18637/jss.v067.i01

**Appendix D – Variation partitioning for community composition models**

Variance partitioning was run for models without block design structure using rdacca.hp package. Significance values from Adonis models with block randomization should be considered. Significant factors are given in bold.

| Soil mites | | | | |
| --- | --- | --- | --- | --- |
|  | Unique | Average.share | Individual | I.perc(%) |
| **Wind disturbance** | **0.0115** | **-0.0005** | **0.011** | **12.64** |
| Elevation | 0.0126 | 0.0042 | 0.0168 | 19.31 |
| Aspect | 0.0035 | 0.0001 | 0.0036 | 4.14 |
| Annual precipitation | 0.0114 | 0.0071 | 0.0185 | 21.26 |
| Slope | 0.0036 | 0.0042 | 0.0078 | 8.97 |
| **OM** | **0.0047** | **0.0018** | **0.0065** | **7.47** |
| Geographic zone | 0.011 | 0.0116 | 0.0226 | 25.98 |

| Springtails | | | | |
| --- | --- | --- | --- | --- |
|  | Unique | Average.share | Individual | I.perc(%) |
| **Wind disturbance** | **0.0261** | **-0.0013** | **0.0248** | **9.25** |
| Elevation | 0.0209 | 0.0194 | 0.0403 | 15.04 |
| **Aspect** | **0.0068** | **0.0053** | **0.0121** | **4.51** |
| Annual precipitation | 0.0205 | 0.0189 | 0.0394 | 14.7 |
| Slope | 0.0117 | -0.0015 | 0.0102 | 3.81 |
| **OM** | **0.0101** | **-0.0002** | **0.0099** | **3.69** |
| **Geographic zone** | **0.0972** | **0.0343** | **0.1315** | **49.07** |
